# The use of mass media by mothers and its association with their children’s early development: comparison between urban and rural areas

**DOI:** 10.1186/s12889-023-16137-1

**Published:** 2023-07-07

**Authors:** M Mofizul Islam

**Affiliations:** Department of Public Health, La Trobe University, Office: Room 410; Health Sciences Building 2, Melbourne, VIC 3086 Australia

**Keywords:** Early childhood development, Mass media, Early childhood development index, Multiple indicator cluster survey, UNICEF, Bangladesh

## Abstract

**Background:**

Mass media can play critical roles in influencing parents’ attitudes and practice toward the healthy upbringing of children.

**Objective:**

This study examined the association between the use of five types of mass media among mothers living in rural and urban areas and the early childhood development (ECD) of their children.

**Methods:**

We analysed nationally representative and internationally standardized Multiple Indicator Cluster Survey data collected in 2013 and 2019 in Bangladesh. The ECD was calculated using four domains of development: physical health, literacy-numeracy, learning and social-emotional. Mothers’ use of newspapers/magazines, radio, television, internet and mobile phones was the study factor. We used Poisson regression with robust variance. The dataset included 27,091 children aged three or four years.

**Results:**

Almost 21% of the children were living in urban and 78% in rural areas. Mothers/caretakers of 30% of the children used none, 39% used one, 25% used two, and approximately 6% used three or more of the five types of media. Mobile phones and television were the dominant types of media, both in terms of the number of users and the frequency of use. Overall, 68.87% of the children were on track in terms of their ECD and 31.13% were not. A significantly larger proportion of urban children (74.23%) than rural children (67.47%) were on track in their ECD. The prevalence of children being on track of ECD increases by 4% (aPR 1.04; 95%CI: 1.01–1.06) for each additional media use among women who lived in urban areas and increases by 7% if women live in rural areas. In terms of the individual formats of media, using newspapers, television and internet was found to be significantly associated with the children in rural areas being on track in terms of their ECD. In the urban sample, only radio use was found to be significant.

**Conclusions:**

Targeted and well-designed child development campaigns that are delivered through popular media types are likely to help mothers to take better care of their children.

## Introduction

In developing countries, over 200 million children younger than five years of age experience delays in their early childhood development (ECD) [1]. These delays are often defined as a deviation of development from the normative milestones in cognitive, language, social, emotional and motor functioning [2]. The growing evidence that ECD influences children’s developmental trajectories and life-courses [1, 3] has received considerable attention and prompted calls for investment in identifying specific factors that affect this development. The literature of ECD identifies a range of psychosocial factors as being vital, in addition to some genetic factors. These psychosocial factors mainly include a number of social determinants of health, such as household wealth status, parental education, parenting skills and early childhood education. Another important but relatively less articulated social determinant is mass media, which play significant roles in forming and influencing parents’ attitudes, behaviours and practice toward the health and healthy upbringing of children [4]. Mass media reach large audiences via mass communication and offer health information to parents about child development.

The theories relevant to the use of mass media for public health improvement through behaviour change connect health communication and attitudinal changes through awareness creation [5]. The assumption is that a person’s attitude, whether implicit or explicit, is a critical intervening factor that links exposure to new information and behavioural change. Mass media has the potential to offer much-needed information about parenting [6]. Parents can become informed, conscious and stay in touch with friends and family members. Social marketing is a good example, which is used in many settings to change people’s behaviours by changing their attitudes. Although there are differences among the experts regarding the steps involved in the translation of attitude to behaviour change [7], the literature suggests that mass media can play a crucial role in creating public awareness, changing attitudes, affecting value systems [8–10] and producing at least a modest degree of behavioural change. Since mass media has a broad reach, even small changes in behaviours can have substantial population-level impacts [11]. Several previous studies found significant associations between exposure to mass media and improved health behaviours, including sufficient fluids and treatment with zinc as recommended for the management of diarrhoea in children [12], changes in fertility rates [13], increased use of contraception [14], and better knowledge about child nutrition [15]. Access to mobile phones increases women’s opportunities for healthcare [16, 17] and nutrition services [18], improves population health and well-being and reduces poverty [19]. Although not all media programmes are specifically aimed at behavioural changes, they can nevertheless bring some changes in norms and aspirations [20]. Since some behaviours are embedded in socio-cultural contexts, mass media that broadcast tailored programmes are likely to be successful [15].

In many low- and middle-income countries (LMICs), mass media is used for public health improvements such as safe pregnancies and deliveries, antenatal and postnatal care, and the healthy upbringing of children. Indeed, some media programmes in Bangladesh received considerable attention and helped to bring about substantial changes in delivery and postpartum care, and in essential care for newborns, child nutrition and development [21, 22]. An animated film, entitled *Meena Cartoon*, is a good example of such a campaign. Since the 1990s, in collaboration with the United Nations Children’s Fund (UNICEF), the government of Bangladesh has been broadcasting this cartoon and other films to deliver messages that relate to early childhood education, gender, child health and social inequality through stories about a nine-year-old girl, Meena, who braves all the odds to go to school and fight discrimination against children, particularly girls. This cartoon quickly became very popular and was subsequently screened on television in other South Asian countries. The telecast of this cartoon demonstrated that early childhood development programs, particularly in popular media, which are relatively easily accessible by various groups in societies, can bring behavioural changes among mothers and subsequently improve the overall development of children. Although not all media campaigns become successful, their overall effectiveness is evaluated as “moderate”, with some variations across settings and campaign types [23, 24].

In the research that has been undertaken to assess the relationship between media use and early childhood development, the amount of screen time among children has received considerable attention, particularly in the recent literature [25–28]. However, although the use of mass media among mothers can change knowledge bases, attitudes and behaviours that relate to their children’s care and upbringing, surprisingly little is known about the relationship between the media use of mothers and the ECD of their children. The dearth of globally accepted indicators for child development has, to some extent, hampered the progress of research in this area [3]. UNICEF recently developed and validated an Early Childhood Development Index (ECDI) that measures the overall scores and those for the four domains of development: physical, literacy-numeracy, learning and social-emotional. This index can be used to identify whether children are developmentally on track in each of the four domains and also overall. The ECDI reflects the population-based normative distribution of the developmental statuses [3] of three- and four-year-old children. This index is the first available tool for measuring early childhood development at the population level in LMICs.

Another aspect of the relationship between media use and ECD is the variation in rural and urban areas. With only a few exceptions, urban residents have greater access and opportunities to use health information sources than rural residents do, regardless of their access to mass media [29]. As healthcare facilities are more available and accessible to them, mothers in urban areas are more likely to use these [30], to receive the necessary health information and to feel an obligation to practice healthy behaviours [31]. Also, mothers in urban settings have far more opportunities than those in rural areas to have interpersonal communication with other knowledgeable people [32, 33]. As a result, there is likely to be a difference between urban and rural settings in terms of the knowledge and attitudes of mothers with regard to the upbringing of their children. As mothers in urban areas already have better knowledge bases, the use of mass media will offer them only relatively few benefits. Quite the opposite is true for mothers in rural areas; the benefits of using mass media are likely to be relatively higher. Therefore, the marginal effects of media use should be much higher among the mothers in rural areas than in urban areas. Other factors being equal, this differential effect should, then, be reflected in the association between the use of mass media among mothers and the early childhood development of their children. Therefore, we hypothesize that the use of mass media among mothers in rural areas will have a greater and more positive effect on the ECD of their children than such use among mothers in urban areas has on their children’s development. Using population-representative data from the 2013 and 2019 Bangladesh Multiple Indicator Cluster Survey (MICS), this paper examines whether this hypothesis holds true.

## Methods

### Data

MICS is a nationally representative and internationally standardized household survey developed by UNICEF that primarily captures information about children, women and households in low- and middle-income countries [34, 35]. We used MICS data collected in 2013 and 2019 survey rounds. MICS in Bangladesh followed a two-stage, stratified cluster sampling approach for the selection of the participants. The urban and rural areas within each of the administrative districts in Bangladesh were identified as the main sampling strata. First, from each stratum, a specified number of census enumeration areas (known as primary sampling units) were selected systematically with probability proportional to size. Second, after identification of the potential households within the selected enumeration areas, a random systematic sample of 20 households was drawn from each primary sampling unit [36, 37]. A total of 2,760 primary sampling units were selected in the 2013 round and 3,220 in the 2019 round. Data were collected in face-to-face interviews with household members. Data on children that constitute the outcome variable of this study come from their mothers, or caregivers if mothers do not live in the household or are deceased. The details of the sampling and data collection procedure of MICS can be found here: *Progotir Pathey*, Bangladesh Multiple Indicator Cluster Survey 2019 [36]. The survey protocol and its ethical aspects were approved by a technical committee of the Bangladesh Bureau of Statistics. Informed consent was appropriately obtained from all participants.

### Mass media (study factor)

The exposure variable for the study was the use of mass media among mothers, through newspapers or magazines, radio, television, the internet and mobile phones and involved five questions, one for each of these types of media. With regard to newspapers and magazines, radio and television, the question posed was how frequently mothers or primary caregivers use these media, such as by reading, listening to and watching them. The response options were as follows: not at all, less than once a week (i.e., less than one time per week), at least once a week and almost every day. In terms of their use of the Internet and mobile phones, the participants were asked how frequently they had used these two types over the past three months. The response options were the same as those for the other three media types.

In the literature, the exposure to mass media is measured mainly in three ways: the use of individual media, number of media use and the frequency of the media use [14, 23, 38]. In this study, we used all three approaches. Mothers or caregivers were identified as media users if they responded affirmatively to any of the following three options: less than once a week, at least once a week, or almost every day. Those who responded “not at all” were identified as non-users. Following this approach, we created a dichotomous response for each form of mass media: user (coded with 1) or not (coded with 0). A summary score was then created, which varied from zero (those who responded “not at all” to all five questions) to five (those who used all five types “less than once a week” or more frequently). For instance, if a mother answered “not at all” for newspapers, radio, television and the internet but “less than once a week” or “at least once a week” or “almost every day” for mobile phones, then her total score would be 1 (one). We named this composite the “number of media types”.

For the frequency of the media use, the four response options were coded as follows: not at all = 0, less than once a week = 1, at least once a week = 2 and almost every day = 3, and then they were added up for each participant. Therefore, the total scores varied from 0 (those who responded “not at all” to all five questions) to 15 (those who responded “almost every day” to all five questions). For instance, if a mother or caregiver responded with “less than once a week” to the first question and “almost every day” to the other four questions, then her total score would be 13 [1 + 3 + 3 + 3 + 3]. This composite was called the “frequency of media use”.

### Outcome variable

The early childhood development index (ECDI) was the outcome variable. This index was calculated for each child using UNICEF’s approach of psychometric computation. The development of the ECDI followed a multi-stage approach. The first stage achieved consensus on the conceptual framework for identifying domains and generating items. In stage2, the framework and list of items to measure child developmental outcomes proposed in Stage1 were pilot tested in Jordan and the Philippines. The final stage involved the validation of the ECDI that took place in Kenya. The details of this index can be sourced elsewhere [3]. Briefly, this index is composed of 10 items (questions) covering four domains of early childhood development (Table 1). Children were considered as being on track in their ECD if they were developmentally on track in at least three of these four domains: physical, literacy-numeracy, learning and social-emotional. In the Multiple Indicator Cluster Survey, these questions are asked to the mothers or primary caregivers with regards to children under five years of age.

**Table 1:** Questions in four domains of ECD and psychometric approach to the identification of children being developmentally on track

Four domains	10 questions	Question item on track if the answer is …	Domain developmentally on track if …
Literacy-numeracy	1. Can the child identify or name at least ten letters of the alphabet?	Yes	At least two items on track
	2. Can the child read at least four simple, popular words?	Yes	
	3. Does the child know the name and recognise the symbol of all numbers from 1 to 10?	Yes	
Physical	4. Can the child pick up a small object with two fingers, like a stick or a rock from the ground?	Yes	At least one item on track
	5. Is the child sometimes too sick to play?	No	
Learning	6. Does the child follow simple directions on how to do something correctly?	Yes	At least one item on track
	7. When given something to do, is the child able to do it independently?	Yes	
Social-emotional	8. Does the child get along well with other children?	Yes	At least two items on track
	9. Does the child kick, bite, or hit other children or adults?	No	
	10. Does the child get distracted easily?	No	

### Other covariates

We also included a range of variables that, as per the literature, are known to be associated with ECD and that are available in our dataset [39–42]. The variables are children’s age and sex; nutritional status measured with stunting, wasting and underweight; their attendance to early childhood education; educational status of mothers; place of residence (urban or rural); households’ wealth status (in quintile); the number of siblings in the household; two structural variables namely, districts and communities; whether children are engaged in stimulation activities [42, 43]. Engagement in stimulation activities was measured using the observations on a question that asks whether mothers, fathers or other household members had been involved with their children in any of the following six activities in the past three days: (1) reading books or looking at pictures; (2) telling stories; (3) singing songs; (4) taking the child outside; (5) playing with the child; (6) naming, counting, or drawing with the child. A summary score was created by counting the responses to these six items [43, 44]. Consequently, the total score for parental stimulation varied from zero (engaged in none of these six activities) to six (engaged in all six activities). Parental stimulation was used as a continuous variable. The primary sampling units were considered as communities.

### Analysis used for assessing the association between mass media and ECD

We used descriptive statistics to compare rural and urban settings in terms of exposure variables, outcome variable and other covariates. For this comparison, the chi-squared test was used for categorical variables and the Mann-Whitney test for continuous variables. To examine the associations between number of media use / frequency of media use and early childhood development index, two sets of Poisson regression (with robust variance) models were developed – one set with the number of media use composite and the other set with the frequency of media use composite. We used Poisson regression with robust variance because the odds ratio estimated using logistic regression from a cross-sectional study may significantly overestimate the relative risk when the outcome is common (e.g., prevalence > 10%) [45–47]. The prevalences of ECDI in overall, urban and rural samples are much higher than 10%.

To test the hypothesis, an interaction term developed using the variables *the number of media use* and *residence (urban and rural)* was included in the model that examines the association with the number of media use in urban and rural settings. Similarly, an interaction term using the variables *frequency of media use* and *residence* was used in the model that examines the association with the frequency of media use in urban and rural settings. To examine the association between the type of media use (e.g., Newspaper, Radio, etc.) and ECD, two separate regression models were developed – one for the urban and the other for the rural sample. All models were adjusted for covariates described in the previous section. Regression results were reported as adjusted prevalence ratio (aPR) and 95% Confidence Interval (95% CI). A higher aPR in the association between ECDI and mass media use among mothers in rural areas than in urban areas would support our hypothesis and a statistically significant higher aPR would demonstrate that the positive association is unlikely to happen by chance alone. Survey weight was employed using primary sampling units, stratification and sample weights to account for the complex survey design. Survey weight was applied both in descriptive and regression estimates. We used STATA version 15 for data analysis.

## Results

The dataset included 27,091 children aged three or four years. Almost 21% of the children were residents of urban areas and 79% were from rural areas (Table 2). Almost half of the children were 3-year-old, and slightly more than half (51.81%) of the children were male. Around 53% of children were stunted or wasted or underweight and only 16% ever attended early childhood education programmes. Overall, 68.87% of the children were on track in terms of their ECD and 31.13% were not. Among the children on track in their ECD, 77.71% were living in rural areas compared to 82.88% of those who were not on track. The comparisons between the children on track in their ECD or not in terms of other relevant variables are presented in Table 2. Mothers/caretakers of 30% of the children used none of the five types of media, 39% used one, 25% used two, 5% used three, and the remaining (1.17%) used four or five types. Mobile phones and television were the dominant types of media, both in terms of the number of users and the frequency of use. The details of the use of media among the mothers across types and frequencies are presented in Table 2. Overall, more mothers/caretakers of children who were on the track of ECD used media than those whose children were not on the track.

**Table 2:** Socio-demographic characteristics of children and use of five forms of media by mothers of children in two groups of ECD

Variable	Overall %	ECD on track %	ECD not on track %
**Residence**			
Urban	20.68	22.29	17.12
Rural	79.32	77.71	82.88
**Children’s age in years**			
3	50.24	45.89	59.88
4	49.76	54.11	40.12
**Children’s sex**			
Male	51.81	49.96	55.90
Female	48.19	50.04	44.10
**Malnutrition**^a^			
Yes	53.47	56.52	46.71
No	46.53	43.48	53.29
**Child ever attended early childhood education programme**			
Yes	15.81	18.85	9.09
No	84.19	81.15	90.91
**Mother’s education level**			
None or per-primary	21.04	18.60	26.43
Primary	28.14	25.94	33.01
Secondary	38.05	40.45	32.75
Higher secondary or more	12.77	15.01	7.82
**Wealth quintile of household**			
Lowest	24.41	21.89	29.99
Lower middle	20.49	19.57	22.53
Middle	18.48	18.50	18.45
Higher middle	18.09	18.51	17.15
Highest	18.53	21.54	11.88
**Number of children gave birth by mother (mean)**	2.54	2.46	2.75
**Frequency of reading newspapers or magazines**			
Not at all	89.80	88.37	93.35
Less than once a week	5.14	5.65	3.88
At least once a week	2.75	3.07	1.97
Almost every day	2.31	2.91	0.80
**Frequency of listening to the radio**			
Not at all	96.29	96.23	96.43
Less than once a week	1.52	1.53	1.50
At least once a week	1.15	1.15	1.15
Almost every day	1.04	1.09	0.91
**Frequency of watching television**			
Not at all	44.00	40.19	52.43
Less than once a week	5.65	5.57	5.85
At least once a week	8.13	8.20	8.00
Almost every day	42.21	46.05	33.72
**Mobile phone usage in the past three months**			
Not at all	1.40	1.15	2.12
Less than once a week	4.39	4.11	5.21
At least once a week	16.79	15.22	21.44
Almost every day	77.43	79.51	71.23
**Internet usage in the past three months**			
Not at all	87.14	85.10	93.19
Less than once a week	1.16	1.26	0.88
At least once a week	1.70	2.09	0.53
Almost every day	10.00	11.55	5.39

All percentages are weighted

*ECD* Early Childhood Development

^a^Children who were stunted or wasted or underweight

Table 3 presents the bi-variable associations of media use and other covariates across urban-rural areas of residence. A significantly higher proportion of urban children (74.23%) than of rural children (67.47) were on track in their ECD. Urban mothers used a greater number of media types than rural mothers and also used them more frequently. Malnutrition, which is known to be significant with regard to ECD, was more prevalent in children living in rural areas than in those in urban areas. Attendance at early childhood education programmes was significantly higher among the urban children (17.58%) than rural children (15.02%). In addition, education levels among urban mothers were significantly higher, and they were also significantly more affluent than their rural counterparts (Table 3). On average, rural mothers gave birth to more children (2.45) than urban mothers (2.16).

**Table 3:** Rural-urban comparison of study factor, outcome variable and other covariates

Variable	Urban	Rural	*p*
Early childhood development on track (%)	74.23	67.47	< 0.01
Malnutrition^a^ (%)	40.77	48.90	< 0.01
Children’s sex (male/female) (%)	50.54 / 49.46	51.69 / 48.31	0.19
Child ever attended early childhood education programme (%)	17.58	15.02	< 0.01
**Mother’s education level (%)**			
None or per-primary	12.97	19.90	< 0.01
Primary	23.80	28.53	
Secondary	37.45	40.57	
Higher secondary or more	25.78	10.99	
**Wealth quintile (%)**			
Lowest	8.98	27.28	< 0.01
Lower middle	8.23	23.31	
Middle	11.16	20.54	
Higher middle	21.62	17.69	
Highest	50.02	11.17	
Number of children ever born (frequency)	2.16	2.45	< 0.01
Children’s average age (in years)	3.49	3.50	0.67
Average number of media types used less than once in a week or more (frequency)^b^	1.49	0.98	< 0.01

All percentages are weighted

^a^Children who were stunted or wasted or underweight

^b^For internet and mobile phone, the question refers to the last 3-month

Table 4 presents the association between the two study factors (number of media use and frequency of media use), other covariates and the outcome variable, ECD. A positive and significant association was found between the numbers of media types that the mothers used and the adjusted prevalence ratios (aPR) of the children being on track in their early childhood development. The interaction term developed with the variables *number of media use* and *residence (urban and rural)* is statistically significant. The prevalence of children being on track of ECD increases by 4% (aPR 1.04; 95%CI: 1.01–1.06) for each additional media use among women who lived in urban areas and increases by 7% (1.04 * 1.03) for each additional media use if women live in rural areas. The interaction term developed using the variables *frequency of media use* and *residence (urban and rural)* in the other model is also statistically significant (last column in Table 4). The association between media use and ECD in children remains similar. Those who were four years old were more likely to be on track in their ECD than those who were three years of age. Overall, the prevalence for female children were 9% higher than male children to be on track in terms of their ECD in both models. The prevalence of the children being on track in terms of their ECD were significantly higher among children who had attended early childhood education programmes, and this was true in both models. The numbers and frequencies of parental stimulation activities that had taken place in the previous three days were not significantly associated with children being on track in their ECD (aPR 1.01; 95% CI: 1.01–1.02). Maternal parity (measured by the number of children to which women have given birth) was negatively associated with the ECD being on track.

**Table 4:** Poisson regression with robust variance examining the association between the number of media types use/frequencies of media use and early childhood development

Variables	Unadjusted	Adjusted	
	ECD PR (95% CI)	For number of media use	For frequency of media use
		ECD aPR (95% CI)	ECD aPR (95% CI)
**Number of media used**	1.10 (1.07–1.12)	1.04 (1.01–1.06)	-
**Frequency of media used**	1.04 (1.03–1.04)	-	1.02 (1.01–1.02)
**Residence**			
Urban	1	1	1
Rural	0.95 (0.90–0.99)	0.94 (0.89–0.99)	0.95 (0.90–0.99)
Number of media use*Residence			
Urban	1	1	-
Rural	1.02 (1.00-1.05)	1.03 (1.01–1.06)	-
Frequency of media use*Residence			
Urban	1	-	1
Rural	1.01 (1.00-1.01)	-	1.01 (1.01–1.02)
**Malnutrition**^a^			
No (ref.)	1	1	1
Yes	0.88 (0.87–0.90)	0.94 (0.92–0.96)	0.94 (0.92–0.96)
**Child’s age**			
3 years (ref.)	1	1	1
4 years	1.19 (1.17–1.22)	1.17 (1.14–1.19)	1.17 (1.14–1.19)
**Child’s sex**			
Male (ref.)	1	1	1
Female	1.08 (1.06–1.10)	1.09 (1.06–1.11)	1.09 (1.06–1.11)
**Child ever attended early childhood education programme**			
No (ref.)	1	1	1
Yes	1.24 (1.21–1.26)	1.11 (1.08–1.13)	1.11 (1.08–1.13)
**Parental stimulation activities in the last 3-day**	1.02 (1.02–1.03)	1.01 (1.01–1.02)	1.01 (1.01–1.01)
**Mother’s education level**			
None or per-primary (ref.)	1	1	1
Primary	1.04 (1.01–1.08)	0.99 (0.96–1.03)	0.99 (0.96–1.03)
Secondary	1.20 (1.17–1.24)	1.07 (1.04–1.11)	1.07 (1.04–1.11)
Higher secondary or more	1.33 (1.29–1.37)	1.10 (1.05–1.14)	1.10 (1.05–1.14)
**Wealth quintile**			
Lowest (ref.)	1	1	1
Lower middle	1.07 (1.03–1.10)	1.02 (0.98–1.05)	1.01 (0.98–1.05)
Middle	1.12 (1.08–1.15)	1.03 (1.00-1.07)	1.03 (0.99–1.06)
Higher middle	1.14 (1.11–1.18)	1.01 (0.98–1.05)	1.00 (0.97–1.04)
Highest	1.30 (1.26–1.33)	1.08 (1.04–1.13)	1.07 (1.03–1.12)
**Number of children ever born**	0.96 (0.95–0.97)	0.98 (0.97–0.99)	0.98 (0.97–0.99)
**Constant**	-	0.58 (0.54–0.62)	0.58 (0.54–0.62)

*PR* unadjusted prevalence ratio; *aPR *adjusted prevalence ratio; ∆ - Adjusted model for number of media use

^a^Children who were stunted or wasted or underweight

§ - Adjusted model for frequency of media use

**Fig. 1:**
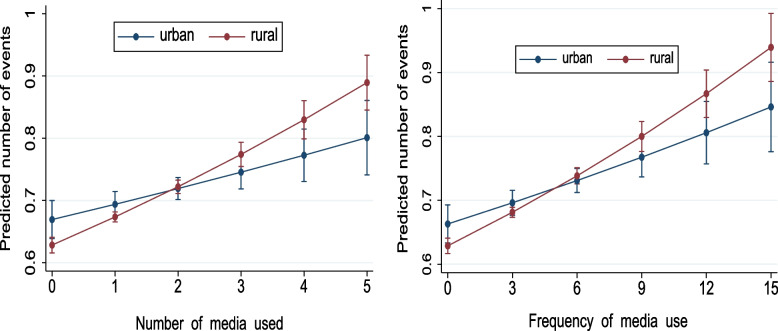
Predicted probabilities of being on track in early childhood development for children living in urban and rural areas

Figure 1 presents the marginal predicted number of events (i.e., ECD on track) for the children living in rural and urban areas, measured in terms of the probability of being on track in ECD for per unit increase in number and frequency of media use while keeping the other variables constant. The slope for the line presenting the probability of rural children being on track in ECD is significantly higher than the corresponding slope for the line presenting urban children. The predicted probabilities for the rural line surpass the urban line as the number and frequency of media use increase (Fig. 1).

Poisson regression outputs of the association between type of media use and ECD are presented in Table 5. In terms of the individual formats of media, in the adjusted model, using newspapers (aPR 1.09; 95%CI 1.04–1.15), television (aPR 1.06, 95%CI 1.02–1.10) and internet (AOR 1.11, 95%CI 1.07–1.16) was found to be significantly and positively associated with the children in rural areas being on track in terms of their ECD. In the adjusted model, there was an elevated association with regard to the mobile phone, but this was not significant, and the aPR for radio use was 0.97 (95%CI 0.87–1.09). In the urban sample, only radio (aPR 1.16, 95%CI 1.09–1.23) was found to be significant. Overall, both unadjusted and adjusted regressions indicate that the likely effect of mass media use by mothers/caregivers on their children’s early childhood development is much higher in rural areas than in urban areas.

**Table 5:** Poisson regression with robust variance examining the associations between media types and early childhood development

Variables	Urban		Rural	
	Unadjusted PR (95% CI)	Adjusted aPR (95% CI)	Unadjusted PR (95% CI)	Adjusted aPR (95%CI)
**Newspaper/magazine**	1.18 (1.13–1.23)	1.01 (0.94–1.10)	1.12 (1.08–1.16)	1.09 (1.04–1.15)
**Radio**	1.00 (0.89–1.13)	1.16 (1.09–1.23)	1.02 (0.96–1.08)	0.97 (0.87–1.09)
**Television**	1.19 (1.11–1.27)	0.93 (0.85–1.02)	1.15 (1.13–1.18)	1.06 (1.02–1.10)
**Mobile phone**	1.07 (0.80–1.42)	0.98 (0.73–1.31)	1.24 (1.05–1.47)	1.16 (0.98–1.37)
**Internet**	1.14 (1.08–1.21)	1.03 (0.97–1.10)	1.20 (1.16–1.24)	1.11 (1.07–1.16)

*aPR* adjusted prevalence ratio; The aPRs for the covariates are now shown

## Discussion

The results of this study suggest that there is a significant association between the use of mass media by mothers in rural areas and their children’s development and that the likelihood of being on track in their ECD increases with the media types used by their mothers. This association is also significant for children in urban areas but with a smaller effect than among children in rural areas. These findings are consistent in terms of both the numbers of media types and the frequencies of media use, and they therefore support our hypothesis. The other important findings are that female children or children who were four years of age or attended early childhood education programmes received parental stimulation activities or lived in households with fewer siblings were significantly more likely than others to be on track in their ECD. To our knowledge, this is the first study that has examined, within an LMIC context, the media use among mothers and the early childhood development of their offspring.

Although the exact reasons for the associations that were observed warrant further research, it is likely that mothers who use mass media have avenues to be informed, sensitised, and acted on better parenting that subsequently help in the early development of their children. Indeed, mass media is a social determinant of health [48]. As we mentioned in the previous sections, the popular cartoon serial, Meena, which was telecast on Bangladesh Television is a good example of how media can play a critical role in child development. Either significant or elevated associations of all the media types, except radio in rural areas, are consistent with our hypothesis. The significant association with radio in urban areas is reasonable, as it is not a popular media type in rural areas, and most radio stations are urban-based and attract members of the younger generations [49]. We recommend further research to examine the reasons why the association with television is not significant.

In Bangladesh, the reach of communication media has increased substantially in the past few decades. Most homes have televisions, radios or mobile phones. According to the latest estimate of 2021, the total number of mobile phone subscribers is now around 170.50 million, and 110.27 million of them have access to the internet [50]. Also, in the past few years, the number of television and radio channels has grown dramatically. As a result, access to media is not as much of an issue now as it was in the past, and we believe that the market force and digital revolution will make the media more available in the next few years if the government is able to provide the basic infrastructure. Therefore, effective use of these media to attain behavioural changes in parenting toward healthy early childhood development should be a top priority for the public health organizations that are concerned with the issue. Indeed, the socio-demographic and educational statuses of the population warrant well-designed and targeted mass media campaigns. The literature suggests that the effectiveness of such campaigns can vary across several variables, such as types, durations, target groups, settings and content quality [10]. Early childhood development programmes are likely to attract attention, not only because it is important but also because it is perceived as a priority by parents and societies. Government, non-government, and international organisations can use this opportunity and target parents and other family members to bring positive behavioural changes towards child development. Since mass media is expanding rapidly in Bangladesh, and the government has initiated several programmes in eHealth (electronic health) and mHealth (mobile health) [51], well-designed media campaigns for early childhood development in popular media such as television, internet and newspapers in rural areas and radio and newspapers in urban are likely to be effective.

A few studies that have been conducted on this topic are mainly in developed countries. Those studies have found that parental use of media technology may interrupt the patterns of interaction between children and their parents. This interruption, sometimes identified as technoference, may have negative effects on early childhood development [52, 53], with parents being slower, less attentive, and more passive in reacting to their children [54, 55], although a recent study found that high levels of media use among mothers are not directly related to their children’s developmental outcomes [25]. It should be noted that in comparison to regular and normal use of the media, excessive use often contributes to inadequate interaction between mothers and their children [25, 55]. Also, excessive use of media is linked to affordability and accessibility, both of which are frequently limited among mothers in rural settings and, more specifically, in those living in resource-poor settings. However, if interactions between mothers and their children and early childhood development are affected by the use of media, our results for urban mothers may have been somewhat affected by this as well, since a subset of the urban mothers may have used media at high levels.

### Strengths and limitations

This study has several strengths and limitations. First, we used a large, nationally representative sample. Second, the ECDI is a validated index than is used consistently in many LMICs. Third, while most previous studies of this topic examined only the overall ECDI, we also examined the four domains that constitute the overall ECDI. Fourth, we used multivariable Poisson regression, and therefore our results are likely to be precise and reliable. However, this is a cross-sectional study, meaning that the associations are associational only. In addition, we could not account for the media use among children or for the use of social media among mothers, as these pieces of information are not available in the dataset. Also, there was a slight difference in the structure of our questions for the media types. Questions about mobile phones and the Internet referred to use over the past three months, whereas the other three types had no time references. Moreover, although the internet and mobile phones are separate types of media, the internet could be accessed using mobile phones. Furthermore, the data represent the situation in only one country. Much of what people receive from mass media with regard to public health is through media campaigns. Therefore, the associations that have been observed are, to some extent, attributable to media campaigns in Bangladesh, and so the results may not be generalizable to other LMICs. We recommend future research examine this association by using a prospective cohort study. Lastly, as the dataset contains only psychosocial variables, we could not account for the biological and genetic endowments of the children in terms of their early childhood developments.

## Conclusions

A higher proportion of urban children than rural children were on track in their ECD. There is a positive association between the number and frequency of mothers’ use of the five media types and the likelihood of their children being on track in their ECD. This positive association is significant for children both in urban and rural areas but with a greater effect in rural areas. Also, there is an increasing trend in these associations, meaning that the proportion of children who are on track in their ECD increases along with the number of media used by their mothers. Children who were four years old; female; attended early childhood education programmes; or received parental stimulation activities were significantly more likely to be on track in their ECD. Conversely, those who were stunted, wasted or underweight; and have a higher number of siblings were less likely to be on track in their ECD. Tailored and innovative mass media campaigns and public health programmes on childhood development and early childhood education are recommended.

## Data Availability

The data that support the findings of this study are publicly available on the website of UNICEF MICS (https://mics.unicef.org/surveys) upon its approval.
